# The Effects of Combined Verbal Encouragement and Technical Instruction on Technical Skills and Psychophysiological Responses During Small-Sided Handball Games Exercise in Physical Education

**DOI:** 10.3389/fpsyg.2022.902088

**Published:** 2022-06-10

**Authors:** Feten Sahli, Raouf Hammami, Hajer Sahli, Nidhal Jebabli, Walid Selmi, Makrem Zghibi, Roland van den Tillaar

**Affiliations:** ^1^Research Unit (Code de l'unité) “Sportive Performance and Physical Rehabilitation”, Higher Institute of Sports and Physical Education of Kef, University of Jendouba, Jendouba, Tunisia; ^2^Higher Institute of Sport and Physical Education of Ksar Said, Manouba University, Tunis, Tunisia; ^3^Research Laboratory (EM2S, UR15JS01) “Education, Motor Skills, Sports and Health”, Higher Institute of Sport and Physical Education of Sfax, University of Sfax, Sfax, Tunisia; ^4^Research Unit (UR17JS01) “Sport Performance, Health & Society”, Higher Institute of Sport and Physical Education of Ksar Said, Manouba University, Tunis, Tunisia; ^5^Department of Sports Science and Physical Education, Nord University, Levanger, Norway

**Keywords:** pupils, motivation, teacher, physical education, performance

## Abstract

To examine the effects of combined positive verbal encouragement and general technical guidelines on technical and psychophysiological parameters in pupils during a small-sided handball passing game. Twenty secondary school pupils (age, 16 ± 1 years; body mass, 55.3 ± 6.6 kg; body height, 1.77 ± 0.13 m; BMI, 22.6 ± 2.2 kg m^−1^) performed small-sided games (2 × 10 min) with three conditions: small-sided game (1) with combined verbal encouragement and technical instruction; (2) with technical instruction; (3) without any instruction (control) in which the passes for each pupil in each set was measured together with the rate of perceived exertion (RPE) and feeling mood after the first and second set. Results: A two-way analysis of variance demonstrated that the combined condition resulted in more passes compared to the technical instruction and control conditions, while the number of passes increased in set 2 for all conditions. RPE and the feeling mood were also differently between the conditions. Combined verbal encouragement and technical instruction during handball exercise were more beneficial for optimal passing numbers, positive mood with less perception of physical effort compared to only technical instruction and a control condition. The results evidenced positive acute effects of combined verbal encouragement and technical instruction during handball, indicating the usefulness of these training methods for optimal passing numbers, positive mood with less perception of physical effort in physical education pupils. Future studies should evaluate the applicability combined verbal encouragement and technical skills in the training of other conditioning capacities and the individual responsiveness of players toward verbal encouragement tasks.

## Introduction

Handball is a strenuous intermittent team sport, characterized by high-intensity activities, with specific requirements for technical skills, tactical understanding, and physical performance such as running, sprinting, and jumping, as well as regular throwing, blocking, and pushing between players (Gorostiaga et al., 2006; Hermassi et al., 2011). To start playing handball, it is necessary to learn the basic technical skills like the reception of the ball, dribbling, passing, and shooting. At handball clubs and at school to learn handball or similar team sports, exercises based on the small games with less rules have become the most used to improve the sports initiation of both players and physical education pupils (Dellal et al., 2012; Halouani et al., 2014; Selmi et al., 2017). Several studies have shown the beneficial effects of exercises based on small games on physical, tactical, technical, and mental parameters in a training and a learning context (Sampaio et al., 2014; Selmi et al., 2017; Sahli et al., 2020, 2021).

In addition, success in high-intensity events can be influenced by providing instruction that focuses on the learner's attention externally, i.e., verbal encouragement (Al-Abood et al., 2002; Porter et al., 2010). In this context, adopting verbal instructions that induce an external focus of attention can be helpful to achieve success when performing movement-related tasks (Eccles and Wigfield, 2002).

The small-sided games represent a specific method that improves physiological demands and technical requirements, used in team sports, based on constraints like small spaces, a lower number of players with specific rules adapting to the game objective (Hill-Haas et al., 2011). According to Iacono et al. (2016), the use of small-sided games in physical education has a potential advantage for learning opportunities for children. Indeed, children have more learning opportunities during small-sided games to increase tactics or strategies than participating in full gameplay (Buchheit et al., 2009). Additionally, small-sided games, in handball as an example, can be a useful exercise to increase the number of contacts with the ball for each player, resulting in more dribbles, passes, and shots (Sassi et al., 2005; Köklü et al., 2011; Tallir et al., 2012). This may contribute to better technical skills (Buchheit et al., 2009; Iacono et al., 2015) during physical education and handball training. Moreover, the small-sided games provide pupils with more learning experiences that improve decision-making opportunities that lead to educational success (Iacono et al., 2016).

The interaction between pupil and teacher may also influence the physical education session process and its outcome. In fact, the behavior of the teacher is defined by their methodological, communicative, and integrative strategies (Pulido et al., 2020; Woods et al., 2020). Regarding communication strategies, verbal behavior, such as general technical guidelines and positive verbal encouragement, could affect physical skills and mood in pupils and athletes. Weakley et al. (2019) reported that the use of technical instructions, during anaerobic exercises, allowed extending a high-intensity effort for more time compared to a non-technical instructions condition. Moreover, Hicheur et al. (2020) reported that verbal feedback, given instantly to the athlete by a coach after training, enhances stress and concentration levels and increases the mental load in soccer players.

Furthermore, the direct influences of other psychological factors, such as motivation (i.e., verbal encouragement), related to an enhanced capacity to act or engage in different achievement tasks (Eccles and Wigfield, 2002) are considered mediators on physical, technical, and tactical abilities of athletes, affecting their performance (Mahamud et al., 2005; Weinberg and Gould, 2019). For example, a positive comment such as “Good match today!” and a positive comment such as “Your match today was very good in person-to-person defense” (Lauber and Keller, 2014) elicit very different reactions in players. Jaffri and Saliba (2021) recently showed that providing verbal encouragement resulted in a greater increase in dynamic balance performance among participants with chronic ankle instability (Jaffri and Saliba, 2021). However, to the best of the authors' knowledge, no study has evaluated the effects of verbal encouragement on the sport-specific technical capacities and mood states in handball. Only the study of Sahli et al. (2020) examined the effects of verbal encouragement given by physical education teachers during small-sided soccer games on the psychophysiological responses, mood state, and physical enjoyment of players. Sahli et al. (2020) showed that small-sided games induced higher physiological responses, rate of perceived exertion (RPE), enjoyment and positive mood in pupil players with a verbal encouragement condition. In mathematics education, Brown and Howard (2014) also showed that by verbal encouragement pupil engagement maintains and/or increases regardless of pupil age and math content level.

Furthermore, since a possible repercussion on the specific-sports initiation could be an important factor in didactic of education, the type of task proposed and the age and previous inexperience of the participants (i.e., pupils in physical education) could probably affect their training with a sports initiation objective. As a hypothesis, the combination of verbal encouragement and technical instruction may reflect a greater advantage on the technical and physical skills of the pupil in the physical education sessions. To the authors' knowledge, no studies have determined the combined effect of positive verbal encouragement and general technical guidelines on small-sided passing games of handball in secondary school pupils. This combination, as a hypothesis, may reflect a greater advantage on the technical and physical performance of the pupil in the physical education sessions.

From the previous literature overview, it is evident that the verbal encouragement may be potentially effective methods (ergogenic aid) to improve the sport-specific capacities. Considering that handball is a sport where the repeated maximal high-intensity is an important physical capacity (Gorostiaga et al., 2006; Hermassi et al., 2011), reports investigating the acute effects of a combined both verbal encouragement and technical skills on the technical sport-specific capacities and mood states in handball players will be particularly beneficial. Finally, although verbal encouragement has been evaluated as methods of improving training effectiveness, there is an evident lack of studies where this method is simultaneously examined regarding their concurrent effectiveness in the context of technical sport-specific initiation and mood states in physical education pupils. Therefore, the main purpose of this study was to measure the effects of combined positive verbal encouragement and general technical guidelines on technical and psychophysiological parameters in pupils during a small-sided handball passing game. Based on the relevant literature (Mahamud et al., 2005; Weinberg and Gould, 2019; Sahli et al., 2020), first, we hypothesized that combined verbal encouragement with technical skills stimuli would enhance the sport-specific technical performance and mood in handball players compared to control conditions.

## Materials and Methods

### Study Design

A cross-sectional within-subject design was used to investigate the effect of combined positive verbal encouragement and general technical guidelines on technical and psychophysiological parameters in pupils during a small-sided handball passing game. The present investigation was carried out during the 2020–2021 education season. After a familiarization session, three experimental sessions of a small-sided passing game in handball were performed during physical education hours. Twenty pupils ranged arbitrarily from 5 vs. 5 on a small area (10 m × 10 m). The small-sided passing game sessions were completed after a standardized 15-min warm-up, such as 5 min of running, 5 min of dynamic stretching, and 5 min of ball-practice. The small-sided passing game sessions were separated by at least 48 h and performed at the same time of the day (±1 h). Pupils were asked to follow their normal diet during the time of the study.

### Sample

Twenty male secondary pupils (age, 16 ± 1 year; body mass, 55.3 ± 6.6 kg; body height, 1.77 ± 0.13 m; BMI, 22.6 ± 2.2 kg m^−1^) were involved in this study. These pupils were selected because they were active in physical education (3 h week^−1^) and had no previous competitive experience in handball. Pupils were assigned to perform, in random order, three sessions of a small-sided passing game in handball, based on three conditions: (1) a small-sided passing game with combined verbal encouragement and technical instruction; (2) a small-sided passing game with technical instruction only; and (3) a small-sided passing game without any instruction/feedback (control). All participants gave their written consent after a full explanation of the purpose of the study and the experimental design. The Ethics Committee of the High Institute of Sports and Physical Education of Kef, Tunisia (Research Unit, Sportive Performance & Physical Rehabilitation, S2PR, pr.nr. CNMSS-LR09SEP01) approved the study, and it was performed in accordance with the principles of the Declaration of Helsinki (2013).

### Procedures

The small-sided passing game sessions consisted of two sets of 10 min separated by 2 min of recovery. The players passed the ball among themselves (same group) as many times as possible, while the defensive group tried to steal the ball from the other team. When the defense team captured the ball, the roles swapped. The players had to follow the rules of handball concerning defense and attack and were not allowed to dribble the ball. Each subject's number of passes was recorded. After a small-sided passing game, RPE (Borg CR10, RPE) was used to measure the perceived exertion for each player after testing (Borg, 1998). Additionally, affect was measured using a feeling scale (−5/+5), that measured the affective response to exercise, during the recovery time and after the small-sided passing game, with participants answering the question “How do you feel right now?” (Hardy and Rejeski, 1989).

The verbal encouragement condition consisted of standardized verbally encouraging statements during the exercise for all groups (i.e., “Come on!,” “Good job!” repetition five: “Excellent!”). All verbal encouragement was at a volume a little louder than normal conversation volume. These encouragements were done regularly from the sideline, while the pupils were playing.

During the technical instruction condition, teachers focused on technical comments (i.e., “Movement to create open passing lanes,” “Movement to maintain possession,” “Support the player on the ball,” “Anticipation and cutting the trajectory of passes,” and “Ball recovery with pressing”) to assist the pupils to improve the quality of passes or their defense during the small-sided handball exercise format. During the combined verbal encouragement and technical instruction condition, the teacher focused on the verbal encouragement during each set to motivate pupils, separated by technical instructions during recovery time. In the technical instruction condition, only instructions were given during the recovery time. The control condition consisted of pupils performing the exercise without any type of instruction or encouragement.

### Statistical Analysis

Data were expressed as means and standard deviations (*SD*). Skewness and kurtosis distribution and normality of data were assessed and confirmed using the Shapiro–Wilk test. A two-way analysis of variance (ANOVA) (3 conditions: combined, technical instruction, and control) × 2 times: was performed on a number of passes, RPE, and feeling scale score. When significant differences were observed, the least significant difference (LSD) and *post hoc* tests were used. Effect sizes were classified as trivial ≤ 0.2, small > 0.2–0.6, moderate > 0.6–1.2, large > 1.2–2.0, and very large > 2.0 magnitudes. The level of significance was set at *p* ≤ 0.05. All analyses were carried out using the SPSS 16 for Windows (SPSS, version 21 for Windows. Inc., Chicago, IL, USA). The level of significance was established at *p* ≤ 0.05.

## Results

**Figure 1:**
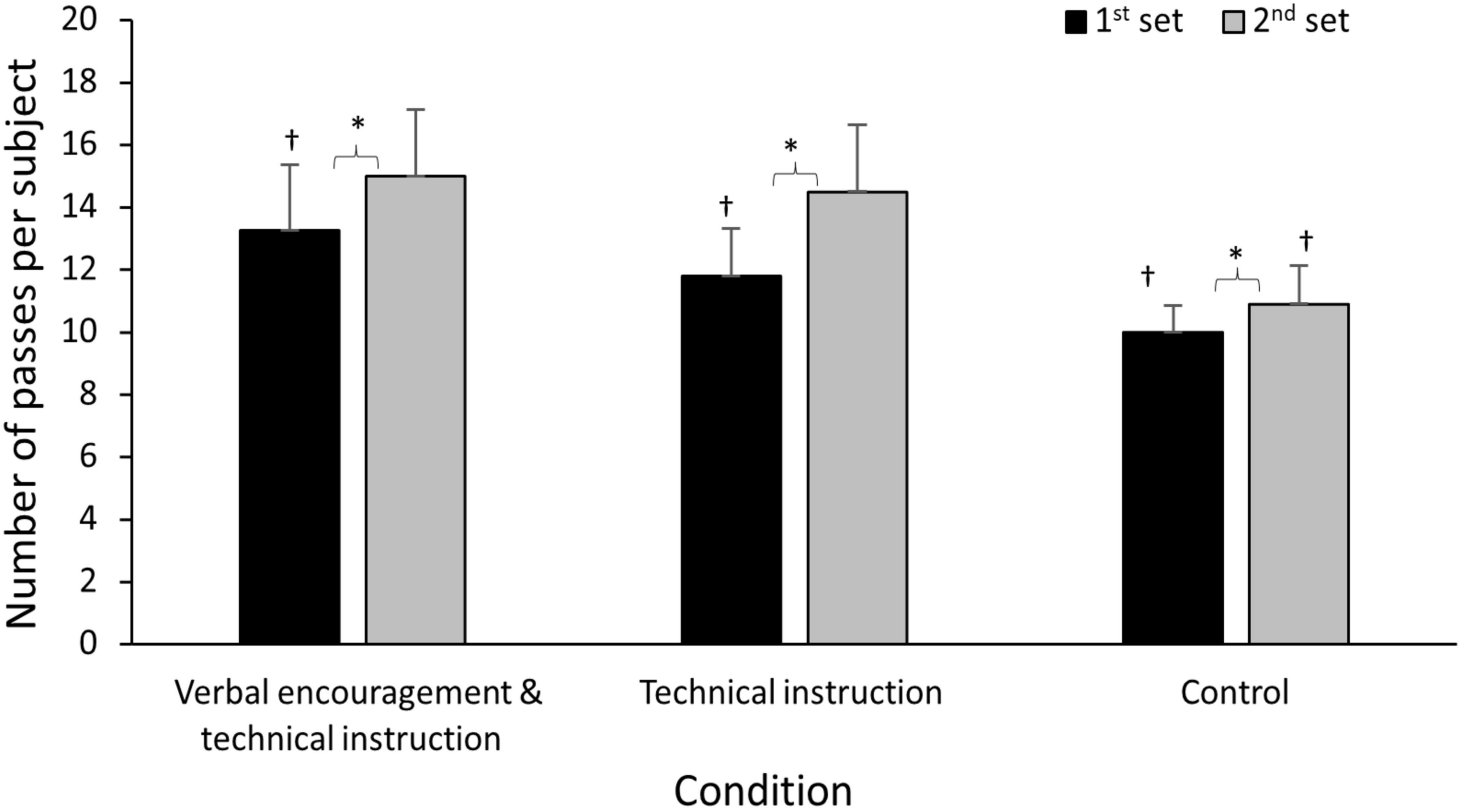
Mean [standard deviation (*SD*)] for number of passes in the first and second set of a small-sided passing game in the three conditions. *indicates a significant difference from 1st to 2nd set at a *p* < 0.05 level. †indicates a significant difference with all other conditions for this set at a *p* < 0.05 level.

For the number of passes, a significant main effect of condition (*F* = 48.6; *p* < 0.001; $\eta_{\text{p}}^{2}$ = 0.72), time effect (*F* = 57.1; *p* < 0.001; $\eta_{\text{p}}^{2}$ = 0.75), and a significant condition ^*^ time interaction (*F* = 10.6; *p* < 0.001; $\eta_{\text{p}}^{2}$ = 0.36) was found. The *post hoc* analysis revealed a significant increase of passing numbers in all three conditions from the first to the second set. During the first set, the number of throws was greatest in the combined condition, followed by the technical instruction and control conditions. However, the technical instruction condition increased most from the first to the second set, followed by the combined and control conditions. This resulted in a similar number of throws between the combined and technical instruction conditions, while the control condition had a significantly lower number of passes (Figure 1).

**Figure 2:**
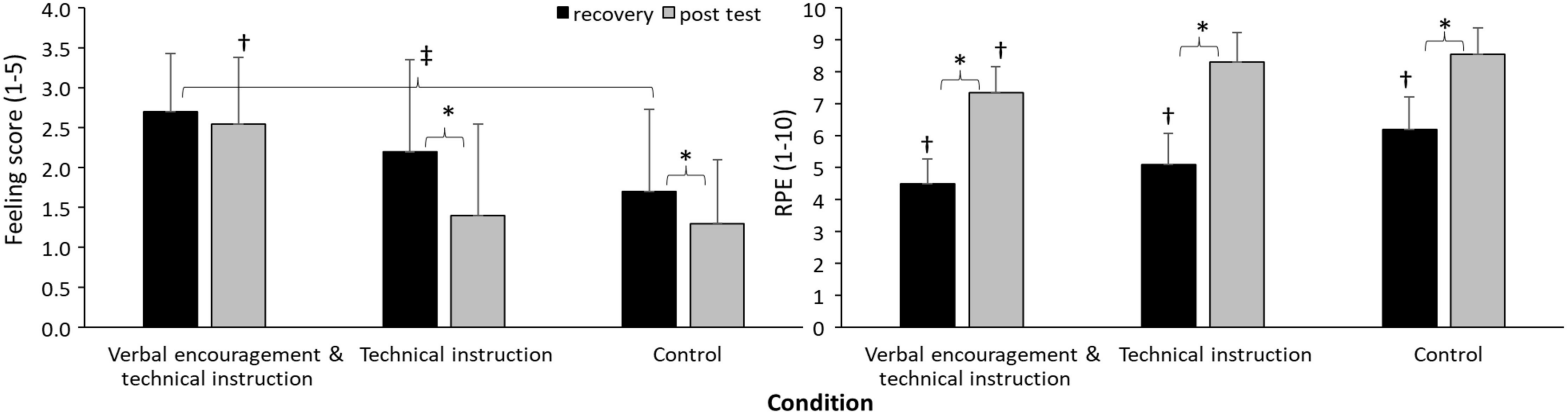
Mean (*SD*) for rate of perceived exertion (RPE) and feeling score under recovery and at post test of a small-sided passing game in the three conditions. *indicates a significant difference from 1st to 2nd set at a *p* < 0.05 level. ^†^indicates a significant difference with all other conditions for this test at a *p* < 0.05 level. ^‡^indicates a significant difference between these two conditions for this test at a *p* < 0.05 level.

For both RPE and feeling scale, a significant effect for condition (*F* ≥ 9.2; *p* < 0.001; $\eta_{\text{p}}^{2}$ = 0.32), time effect (*F* ≥ 16.2; *p* < 0.001; $\eta_{\text{p}}^{2}$ ≥ 0.46), and interaction effect (*F* ≥ 4.5; *p* ≤ 0.018; $\eta_{\text{p}}^{2}$ ≥ 0.19) was found. The *post hoc* comparison revealed that RPE after the first set was significantly lower for the combined condition, followed by the technical instruction and control conditions. The RPE increased for all groups, however, the RPE of the post test of the technical instruction condition increased significantly more than the control group, resulting in similar RPEs at the post test. The feeling score was significantly higher for the combined compared with the control condition during recovery. Furthermore, the feeling score in both the technical instruction and control conditions decreased significantly from recovery to the post test, while no significant difference in feeling score was found in the combined condition (Figure 2).

## Discussion

This study examined the effects of combined verbal encouragement and technical instruction on technical and psychophysiological parameters in pupils during a small-sided passing game in handball. The main findings were that the combined condition resulted in more passes during set 1 and set 2 of a small-sided handball passing game exercise compared to technical instruction and control conditions, while the number of passes increased in set 2 for all conditions. In addition, RPE after each set was significantly lower for the combined condition, followed by the technical instruction compared to control conditions, while the feeling scale was higher for the combined condition during recovery compared with the control condition. After exercise, the feeling score decreased in the technical and control conditions, while it stayed the same in the combined condition.

The number of passes per pupil was higher in the first set during the combined condition compared to the other conditions, indicating that verbal encouragement helped in increasing the number of passes in a small-sided handball passing game exercise. The technical instruction during recovery also helped in increasing the number of passes as shown by the greater increase in number of passes in both the combined and technical instruction conditions compared to the control condition (Figure 1). Initially, *post hoc* testing demonstrated changes with an important increase of passing number, for each pupil, sport initiation skills throughout the combined verbal encouragement and technical instruction conditions. The current results are in agreement with previous studies that have shown beneficial effects of verbal encouragement and technical instruction separately in small-sided games. Sahli et al. (2020) showed that small-sided games, during a verbal encouragement condition, induced higher physiological responses, RPE, enjoyment and positive mood in pupil players. Práxedes et al. (2016) found that the use of technical instruction improved the individual actions of players during a small-sided games exercise, such as the number of passes and the dribble. In addition, Ennes et al. (2008) observed that verbal instruction helped in the process of acquiring motor skills in sports, even if there were multifactorial explanations for the phenomenon. Among those factors, García et al. (2013) showed that technical instructions were most effective on the velocity and precision during handball shots for less experienced players compared to experienced ones. However, when combining verbal encouragement with technical instruction, as done in the present study, it seems to improve technical skills for pupils from the first moment as seen during the first 10-min (set 1) of a small-sided games exercise than technical instruction or verbal encouragement separately.

Although both the combined and technical instruction conditions increased the number of passes from set 1 to 2, the RPE and feeling scale behaved differently between the two conditions: the feeling scale stayed the same and the RPE did not increase as much in the combined condition compared with the technical instruction condition. This indicates that using only technical instructions during recovery adds more stress on the pupils as shown by the lower positive mood and higher perceived exertion after set 2 (Figure 2). The current results were in disagreement with Brandes and Elvers (2017) who reported a decrease in physical load levels with the use of technical instructions during small-sided games, even if the RPE increased. These differences could be due to the coaches' behavior (Foulds et al., 2019), coach encouragement (Teques et al., 2019; Díaz-García et al., 2021), technical information processes (Trigueros et al., 2019), and players' satisfaction (García-Calvo et al., 2014). Another reason for the higher increased RPE in the technical instruction than the combined condition in set 2 may be due to improved passing scores and, therefore, more physical fatigue. As the passing scores in the combined condition were already higher in set 1 than in the technical instruction condition, the increase in passing score did not increase so much and thereby did not increase the RPE as much as during the technical instruction condition.

Regarding the feeling scale, the combined condition kept the positive mood up during the passing game, while it decreased in the other two conditions. To our knowledge, no studies have determined the combined effect of positive verbal encouragement and general technical instructions on the feeling scale. However, our results are consistent with studies that observed an increase of positive mood during small-sided games with the use of verbal encouragement only (Sahli et al., 2020; Sparkes et al., 2020). It seems that by verbal encouragement the passing game is considered as less stressful than when only using technical instructions. The combination gives the pupil new insights by the technical instructions (more passes), but the encouragement during the sets keeps the pupils positive about their sport initiation skills and thereby considering the technical instructions as less stressful. However, no additional analysis by questionnaires was performed that could underline this positive effect of the combined condition. Future studies should include this type of analysis to investigate why the pupils maintained a more positive mood during the combined condition compared with the other conditions. Finally, since we focus on didactic of physical education, the type of task proposed and the age and previous inexperience of the participants (i.e., student in physical education) could probably affect their training with sports initiation skills. This explanation could, therefore, explain the superior changes in outcome in favor of the combined verbal encouragement and technical skills with the present sample.

There are several study limitations. First, the sample of athletes involved comprised youth handball players. Therefore, the results obtained can only be generalized to similar samples of participants. Furthermore, the use of more authentic assessments such as the GPAI instrument to assess sport initiation skills changes in team games such as team basketball (Harvey et al., 2017). These instruments can be used to capture both on-the-ball technical aspects and off-the-ball support movements and thus a more reliable inference as to how the game dynamics influenced the level of exertion that players exhibit during games. In invasion games with larger numbers, e.g., 5v5 involvement with the ball is more limited and the ability to maintain team possession is often dictated by the off-the-ball support which is not captured by the current assessment of “passes.” In addition, by using just the technical aspects of “passing” and attempting to connect it to physiological responses such as RPE, a very important physical aspect of invasion games is recommended for further research. Finally, this study observed sport initiation skill variables and did not measure psychophysiological response variables (i.e., blood lactate, heart rate, and GPS measurement), which could give us more information about the effect of encouragement upon the intensity of the passing game. This will certainly allow us to discuss the obtained results more profoundly. Therefore, although not being the final word on the topic, we believe that the results of this study will improve the knowledge in this field and initiate further research. Furthermore, the sample size was a bit small. Therefore, this study is preliminary. While our results provide interesting information for coaches and strength and conditioning specialists, they have to be interpreted with caution and should be verified in future studies. Finally, with the ages of the participants and their inexperience in handball, it is difficult to consider a passing game as an activity that develops a specific performance technique. Further investigation should be conducted in teams of this sport rather than in physical education classes to investigate if the used didactics also have the same result in a moe sport setting.

## Conclusions

In conclusion, our results showed that combined verbal encouragement and technical instruction improved the passing number in a small-sided handball passing game with a greater RPE score and positive mood scale. Physical education teachers may consider using this combination of verbal encouragement and technical instruction in small-sided passing games to improve the quality of ball flow. It seems that combined verbal encouragement is an effective method to improve pupil engagement during small-sided games exercises. Consequently, in future studies, individual responsiveness to combined verbal encouragement and technical instruction should be evaluated. Such experiments will eventually reveal the background of the combined verbal encouragement and technical instruction-related effects on the studied performances (and individual athletes) and assure proper individualization of the training in youth handball players, at least when it comes to the conditioning of small-sided games in handball is directly related to competitive success, these findings should be translated into regular handball training to benefit a competitive success.

## Data Availability Statement

The raw data supporting the conclusions of this article will be made available by the authors, without undue reservation.

## Ethics Statement

The studies involving human participants were reviewed and approved by the Ethics Committee of the High Institute of Sports and Physical Education of Kef, Tunisia. Written informed consent to participate in this study was provided by the participants' legal guardian/next of kin.

## Author Contributions

FS, HS, RH, MZ, and NJ participated to the conception and design of the study. FS and HS were responsible for testing. FS, HS, NJ, MZ, and RT were responsible for data collection and statistical analysis. RH, FS, HS, WS, and RT were responsible for writing and finalization of the manuscript. All authors contributed to manuscript and approved the submitted version.

## Conflict of Interest

The authors declare that the research was conducted in the absence of any commercial or financial relationships that could be construed as a potential conflict of interest.

## Publisher's Note

All claims expressed in this article are solely those of the authors and do not necessarily represent those of their affiliated organizations, or those of the publisher, the editors and the reviewers. Any product that may be evaluated in this article, or claim that may be made by its manufacturer, is not guaranteed or endorsed by the publisher.

## References

[B1] Al-Abood S. A. Bennett S. J. Hernandez F. M. Ashford D. Davids K. (2002). Effect of verbal instructions and image size on visual search strategies in basketball free throw shooting. J Sports Sci 20, 271–278. 10.1080/026404102317284817 11999481

[B2] Borg G. (1998). Borg's Perceived Exertion and Pain Scales. Champaign, IL: Human Kinetics.

[B3] Brandes M. Elvers S. (2017). Elite youth soccer players' physiological responses, time-motion characteristics, and game performance in 4 vs. 4 small-sided games: the influence of coach feedback. J. Strength. Cond. Res. 31, 2652–2658. 10.1519/JSC.0000000000001717 28933710

[B4] Brown L. N. Howard A. M. (2014). The positive effects of verbal encouragement in mathematics education using a social robot, in: 2014 IEEE Integrated STEM Education Conference: IEEE, 1–5. 10.1109/ISECon.2014.6891009

[B5] Buchheit M. Laursen P. Kuhnle J. Ruch D. Renaud C. Ahmaidi S. (2009). Game-based training in young elite handball players. Int. J. Sports Med. 30, 251–258. 10.1055/s-0028-1105943 19199207

[B6] Dellal A. Owen A. Wong D. Krustrup P. Van Exsel M. Mallo J. (2012). Technical and physical demands of small vs. large sided games in relation to playing position in elite soccer. Hum. Mov. Sci. 31, 957–969. 10.1016/j.humov.2011.08.013 22341858

[B7] Díaz-García J. Pulido J. J. Ponce-Bordón J. C. Cano-Prado C. López-Gajardo M. Á. García-Calvo T. (2021). Coach encouragement during soccer practices can influence players' mental and physical loads. J. Hum. Kinet. 79, 277–288. 10.2478/hukin-2021-0079 34401006 PMC8336561

[B8] Eccles J. S. Wigfield A. (2002). Motivational beliefs, values, and goals. Ann. Rev. Psychol. 53, 109–132. 10.1146/annurev.psych.53.100901.135153 11752481

[B9] Ennes F. C. M. Bruzi A. T. Vieira M. M. Dutra L. N. Ugrinowitsch H. Benda R. N. (2008). A demonstração ea instrução verbal na aquisição de habilidades esportivas. Rev. Min. Educ. Fís. 16, 108–133.

[B10] Foulds S. J. Hoffmann S. M. Hinck K. Carson F. (2019). The coach–athlete relationship in strength and conditioning: high performance athletes' perceptions. Sports 7, 244. 10.3390/sports7120244 31817157 PMC6956380

[B11] García J. A. Sabido R. Barbado D. Moreno F. J. (2013). Analysis of the relation between throwing speed and throwing accuracy in team-handball according to instruction. Eur. J. Sports Sci. 13, 149–154. 10.1080/17461391.2011.606835

[B12] García-Calvo T. Leo F. M. Gonzalez-Ponce I. Sánchez-Miguel P. A. Mouratidis A. Ntoumanis N. (2014). Perceived coach-created and peer-created motivational climates and their associations with team cohesion and athlete satisfaction: evidence from a longitudinal study. J. Sports Sci. 32, 1738–1750. 10.1080/02640414.2014.918641 24911047

[B13] Gorostiaga E. M. Granados C. Ibañez J. González-Badillo J. J. Izquierdo M. (2006). Effects of an entire season on physical fitness changes in elite male handball players. Med. Sci. Sports Exerc. 38, 357–366. 10.1249/01.mss.0000184586.74398.03 16531907

[B14] Halouani J. Chtourou H. Gabbett T. Chaouachi A. Chamari K. (2014). Small-sided games in team sports training: a brief review. J. Strength. Cond. Res. 28, 3594–3618. 10.1519/JSC.0000000000000564 24918302

[B15] Hardy C. J. Rejeski W. J. (1989). Not what, but how one feels: the measurement of affect during exercise. J. Sport Exerc. Psychol. 11, 304–317. 10.1123/jsep.11.3.304

[B16] Harvey S. Gil-Arias A. Smith M. L. Smith L. R. (2017). Middle and elementary school students' changes in self-determined motivation in a basketball unit taught using the tactical games model. J. Hum. Kinet. 59, 39–53. 29134047 10.1515/hukin-2017-0146 PMC5680685

[B17] Hermassi S. Chelly M. S. Tabka Z. Shephard R. J. Chamari K. (2011). Effects of 8-week in-season upper and lower limb heavy resistance training on the peak power, throwing velocity, and sprint performance of elite male handball players. J. Strength Cond. Res. 25, 2424–2433. 10.1519/JSC.0b013e3182030edb 21869628

[B18] Hicheur H. Chauvin A. Cavin V. Fuchslocher J. Tschopp M. Taube W. (2020). Augmented-feedback training improves cognitive motor performance of soccer players. Med. Sci. Sports Exerc. 52, 141–152. 10.1249/MSS.0000000000002118 31425382

[B19] Hill-Haas S. V. Dawson B. Impellizzeri F. M. Coutts A. J. (2011). Physiology of small-sided games training in football: a systematic review. Sports Med. 41, 199–220. 10.2165/11539740-000000000-00000 21395363

[B20] Iacono A. D. Ardigò L. P. Meckel Y. Padulo J. (2016). Effect of small-sided games and repeated shuffle sprint training on physical performance in elite handball players. J. Strength Cond. Res. 30, 830–840. 10.1519/JSC.0000000000001139 26907846

[B21] Iacono A. D. Eliakim A. Meckel Y. (2015). Improving fitness of elite handball players: small-sided games vs. high-intensity intermittent training. J. Strength Cond. Res. 29, 835–843. 10.1519/JSC.0000000000000686 25226326

[B22] Jaffri A. H. Saliba S. (2021). Does verbal encouragement change dynamic balance? The effect of verbal encouragement on Star Excursion Balance Test performance in chronic ankle Instability. Braz. J. Phys. Ther. 25, 617–622. 10.1016/j.bjpt.2021.04.002 34001425 PMC8536856

[B23] Köklü Y. Asçi A. Koçak F. Ü. Alemdaroglu U. Dündar U. (2011). Comparison of the physiological responses to different small-sided games in elite young soccer players. J. Strength Cond. Res. 25, 1522–1528. 10.1519/JSC.0b013e3181e06ee1 21399538

[B24] Lauber B. Keller M. (2014). Improving motor performance: selected aspects of augmented feedback in exercise and health. Eur. J. Sports Sci. 14, 36–43. 10.1080/17461391.2012.725104 24533493

[B25] Mahamud J. Tuero C. Márquez S. (2005). Características psicológicas relacionadas con el rendimiento: comparación entre los requerimientos de los entrenadores y la percepción de los deportistas. Rev. Psicol. Dep. 14, 237–251.

[B26] Porter J. Nolan R. Ostrowski E. Wulf G. (2010). Directing attention externally enhances agility performance: a qualitative and quantitative analysis of the efficacy of using verbal instructions to focus attention. Front. Psychol. 1, 216. 10.3389/fpsyg.2010.00216 21833271 PMC3153821

[B27] Práxedes A. Moreno A. Sevil J. García-González L. Del Villar F. (2016). A preliminary study of the effects of a comprehensive teaching program, based on questioning, to improve tactical actions in young footballers. Percept. Mot. Skills 122, 742–756. 10.1177/0031512516649716 27207601

[B28] Pulido C. M. Ruiz-Eugenio L. Redondo-Sama G. Villarejo-Carballido B. (2020). A new application of social impact in social media for overcoming fake news in health. Int. J. Environ. Res. Pub. Health 17, 2430. 10.3390/ijerph17072430 32260048 PMC7177765

[B29] Sahli H. Haddad M. Jebabli N. Sahli F. Ouergui I. Ouerghi N.. (2021). The effects of verbal encouragement and compliments on physical performance and psychophysiological responses during the repeated change of direction sprint test. Front. Psychol. 12, 698673–698673. 10.3389/fpsyg.2021.698673 35250684 PMC8896045

[B30] Sahli H. Selmi O. Zghibi M. Hill L. Rosemann T. Knechtle B.. (2020). Effect of the verbal encouragement on psychophysiological and affective responses during small-sided games. Int. J. Environ. Res. Pub. Health 17, 8884. 10.3390/ijerph17238884 33260395 PMC7731112

[B31] Sampaio J. E. Lago C. Gonçalves B. Maçãs V. M. Leite N. (2014). Effects of pacing, status and unbalance in time motion variables, heart rate and tactical behaviour when playing 5-a-side football small-sided games. J. Sci. Med. Sport. 17, 229–233. 10.1016/j.jsams.2013.04.005 23683687

[B32] Sassi R. Reilly T. Impellizzeri F. (2005). A comparison of small-side games and interval training in elite professional soccer players. Sci. Football V, 352–354.

[B33] Selmi O. Haddad M. Majed L. Khalifa B. Hamza M. Chamari K. (2017). Soccer training: high-intensity interval training is mood disturbing while small sided games ensure mood balance. J. Sports Med. Phys. Fitn. 58, 1163–1170. 10.23736/S0022-4707.17.07292-9 28488826

[B34] Sparkes W. Turner A. N. Weston M. Russell M. Johnston M. Kilduff L. P. (2020). The effect of training order on neuromuscular, endocrine and mood response to small-sided games and resistance training sessions over a 24-h period. J. Sci. Med. Sport. 23, 866–871. 10.1016/j.jsams.2020.01.017 32061525

[B35] Tallir I. Philippaerts R. Valcke M. Musch E. Lenoir M. (2012). Learning opportunities in 3 on 3 versus 5 on 5 basketball game play: an application of nonlinear pedagogy. Int. J. Sport Psychol. 43, 420–437.

[B36] Teques P. Duarte D. Viana J. (2019). Coaches' emotional intelligence and reactive behaviors in soccer matches: mediating effects of coach efficacy beliefs. Front. Psychol. 10, 1629. 10.3389/fpsyg.2019.01629 31379667 PMC6647934

[B37] Trigueros R. Aguilar-Parra J. M. López-Liria R. Rocamora P. (2019). The dark side of the self-determination theory and its influence on the emotional and cognitive processes of students in physical education. Int. J. Environ. Res. Pub. Health 16, 4444. 10.3390/ijerph16224444 31726790 PMC6888335

[B38] Weakley J. J. Read D. B. Fullagar H. H. Ramirez-Lopez C. Jones B. Cummins C.. (2019). How am i going, coach?—the effect of augmented feedback during small-sided games on locomotor, physiological, and perceptual responses. Int. J. Sports Physiol. Perf. 15, 677–684. 10.1123/ijspp.2019-0078 31715583

[B39] Weinberg R. S. Gould D. (2019). Foundations of Sport and Exercise Psychology, 7th Edn, Champaign, IL: Human Kinetics.

[B40] Woods J. A. Hutchinson N. T. Powers S. K. Roberts W. O. Gomez-Cabrera M. C. Radak Z.. (2020). The COVID-19 pandemic and physical activity. Sports Med. Health. Sci. 2, 55–64. 10.1016/j.smhs.2020.05.006 34189484 PMC7261095

